# Comparison of indocyanine green and blue-stained glue for preoperative localization for pulmonary nodules

**DOI:** 10.3389/fonc.2024.1345288

**Published:** 2024-03-21

**Authors:** Jia Lin, Jia Zhang, Ning Wei, An-Le Wu, Long-Fei Wang, Fei Teng, Yu-Tao Xian, Rui Han

**Affiliations:** ^1^ Department of Interventional Radiology, The First Affiliated Hospital of Ningbo University, Ningbo, China; ^2^ Department of Radiology, Xuzhou Central Hospital, Xuzhou, China; ^3^ Department of Interventional Radiology, The First Affiliated Hospital of University of Science and Technology of China (Anhui Provincial Hospital), Hefei, China; ^4^ Department of Thoracic Surgery, The First Affiliated Hospital of Ningbo University, Ningbo, China

**Keywords:** indocyanine green, blue-stained glue, localization, pulmonary nodule, computed tomography, video-assisted thoracic surgery, ground glass nodule, solid nodule

## Abstract

**Background:**

In patients with pulmonary nodules undergoing computed tomography (CT)-guided localization procedures, a range of liquid-based materials have been employed to date in an effort to guide video-assisted thoracoscopic surgery (VATS) procedures to resect target nodules. However, the relative performance of these different liquid-based localization strategies has yet to be systematically evaluated. Accordingly, this study was developed with the aim of examining the relative safety and efficacy of CT-guided indocyanine green (IG) and blue-stained glue (BSG) PN localization.

**Methods:**

Consecutive patients with PNs undergoing CT-guided localization prior to VATS from November 2021 - April 2022 were enrolled in this study. Safety and efficacy outcomes were compared between patients in which different localization materials were used.

**Results:**

In total, localization procedures were performed with IG for 121 patients (140 PNs), while BSG was used for localization procedures for 113 patients (153 PNs). Both of these materials achieved 100% technical success rates for localization, with no significant differences between groups with respect to the duration of localization (P = 0.074) or visual analog scale scores (P = 0.787). Pneumothorax affected 8 (6.6%) and 8 (7.1%) patients in the respective IG and BSG groups (P = 0.887), while 12 (9.9%) and 10 (8.8%) patients of these patients experienced pulmonary hemorrhage. IG was less expensive than BSG ($17.2 vs. $165). VATS sublobar resection procedure technical success rates were also 100% in both groups, with no instances of conversion to thoracotomy.

**Conclusions:**

IG and BSG both offer similarly high levels of clinical safety and efficacy when applied for preoperative CT-guided PN localization, with IG being less expensive than BSG.

## Introduction

Chest computed tomography (CT) scans frequently detect pulmonary nodules (PNs) in evaluated patients, prompting a need for further investigation (1–3). Video-assisted thoracic surgery (VATS) is a diagnostic complementary tool for pathological diagnosis of the PNs when both CT-guided and bronchoscopic biopsy strategies have failed prior to VATS as this procedure can provide accurate pathological insight and may even be curative in some instances (4–6). To facilitate accurate PN resection, it is essential that target nodules be effectively localization prior to VATS resection, particularly in cases of small nodules or ground glass nodules (GGNs) (7).

CT-guided preoperative PN localization can reduce the odds of conversion to thoracotomy on subsequent VATS-based nodule resection (8). A wide array of localization materials have been designed to date, with the most common being coil, hook-wire, and liquid materials. In recent meta-analyses, liquid-based localization procedures have been linked to higher rates of successful localization relative to coil- or hook-wire-based procedures while also reducing the rates of perioperative complications (9, 10). These liquid materials can be easily applied to facilitate PN localization, and a large variety of materials have been developed for this purpose to date including indocyanine green (IG), medical glue, methylene blue (MB), and iodipin (9). Few studies to date, however, have sought to compare the relative efficacy of different liquid-based localization strategies.

Here, the relative safety and performance of CT-guided preoperative PN localization procedures performed using IG and blue-stained glue (BSG) were compared.

## Methods

### Study design

The Ethics Committee of The First Affiliated Hospital of Ningbo University (No. 2023-080A-02) and Anhui Provincial Hospital (No. 2022-RE-269) approved this retrospective analysis of patients from two centers, and the requirement for informed consent was waived.

This study analyzed data from consecutive patients with PNs undergoing preoperative CT-guided localization procedures performed using BSG or IG prior to VATS resection from November 2021 - April 2022. BSG localization was conducted in Anhui Provincial Hospital and IG was conducted in The First Affiliated Hospital of Ningbo University. Patients eligible for inclusion were: (1) individuals with PNs, (2) individuals facing a high risk of malignancy as determined through clinical and radiological examination (11), and (3) patients for whom preoperative PN localization was required. Patients were excluded if they: (1) exhibited PNs < 5 mm in diameter, (2) harbored calcified PNs, or (3) harbored PNs that decreased in size on CT-based follow-up.

### Preoperative analyses

Chest CT scans for all patients were conducted before localization procedures. Localization was indicated in patients with: (1) GGNs or (2) solid PNs ≤ 15 mm in diameter. CT results were used to determine the characteristics, diameter, location, and PN-pleura distance for each target nodule.

### CT-guided IG localization

A 16-row CT machine (Siemens, Berlin, Germany) was utilized for IG (25 mg, Dandong Yichuang Pharmaceutical Co. Ltd, Liaoning, China) localization, which was performed under local anesthesia using the following settings: 120 Kv tube voltage, 100 mA tube current, 2 mm thickness, 0.6 second gantry rotation time, and 1.1 pitch.

**Figure 1:**
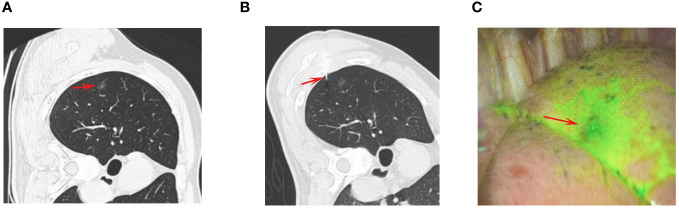
The procedures of CT-guided IG localization. **(A)** The chest CT showed a GGN (arrow) at the right upper lobe. **(B)** The puncture needle (arrow) was sent into the lung and near the GGN for IG localization. **(C)** The IG (arrow) could be visualized during the VATS procedure.

The locations of target nodules were used to determine patient positioning, puncture sites, and needle pathways (**Figure 1A**). Needle pathway selection was performed so as to avoid any fissures, bronchi, large vessels, or emphysema while minimizing the PN-pleura distance to the greatest extent possible. After inserting a 21G introducer needle (Argon Medical Devices, Inc, TX, USA) into the lung parenchyma, repeated CT scanning was performed to enable adjustment of the needle until it’s the tip of the needle was within 10 mm of the target PN (**Figure 1B**). Following CT-based confirmation that the needle was optimally positioned, ~0.15 mL of IG was gradually injected while slowly withdrawing the needle such that IG remained visible on the visceral pleura.

### CT-guided BSG localization

**Figure 2:**
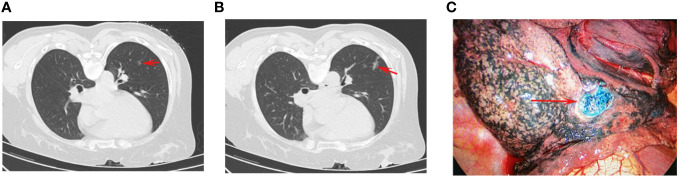
The procedures of CT-guided BSG localization. **(A)** The chest CT showed a GGN (arrow) at the left lower lobe. **(B)** The BSG (arrow) was injected near the GGN for localization. **(C)** The BSG (arrow) could be visualized during the VATS procedure.

BSG was prepared by mixing MB (2 mL: 20 mg, JumpCan Pharmaceutical, Jiangsu, China) with medical glue (1 mL/ampoule, Fuaile Co. Ltd, Beijing, China). Initially, a 1 mL syringe was used to collect 0.2 mL of glue, after which it was mixed with 0.05 mL of MB. As medical glue coagulates relatively quickly, BSG was only prepared after the puncture needle was already positioned appropriately. The remainder of the CT-guided puncture and localization procedures for these patients were the same as those for patients undergoing IG-based localization (**Figures 2A, B**). After optimal needle positioning had been confirmed, ~0.2 mL of BSG was rapidly injected through the needle, which was subsequently withdrawn.

### Post-localization assessment

Localization-associated complications were assessed via chest CT examination in all cases.

### VATS procedures

VATS procedures were performed within 3 h following localization for all patients under general anesthesia using a standardized two-incision approach. In IG group, the VATS was performed with an infrared fluorescent thoracoscope (Novadaq, Mississauga, Canada). In BSG group, the VATS was performed with a normal thoracoscope (KARL STORZ, Tuttlingen, Germany). The thorascopic port was placed on the midaxillary line in the 7^th^ intercostal space, while the operative port was placed on the anterior axillary line in the 4^th^ intercostal space. Wedge or segmental sublobar resection was then performed based on IG or BSG visualization (**Figures 1C**, **2C**). The intra-operative identification of the PNs was made by combination of the visualization of localization markers, intra-operative palpation, and referring the preoperative CT results. For wedge resection, the resected range was at least 2 cm from the localized area, with segmental resection being conducted in cases where wedge resection could not ensure adequate margins. Intraoperative pathological frozen section analysis was performed for all resected tissue samples in the Department of Pathology. No follow-up procedures were immediately performed for patients with benign PNs, precancerous lesions, or diagnoses of cancer *in situ*, mini-invasive cancer, or metastatic lesions. When resected PNs were diagnosed as invasive tumors, subsequent lobectomy and lymph node dissection were recommended.

### Definitions

CT-guided localization procedures were deemed a technical success when IG/BSG were visible on the surface of the lung without any diffusion away from the site of injection. VATS procedures were considered a technical success when the target PN could be identified within the resected portion of pulmonary parenchymal tissue. Pulmonary hemorrhage was defined by evidence of new-onset consolidating/ground-glass opacity around the needle tract > 2 cm in width (12). Localization-related pain was assessed with a visual analog scale (VAS).

### Statistical analyses

Quantitative data were respectively given as means ± standard deviations or medians when normally distributed and skewed, and were compared using Student’s t-tests or Mann-Whitney U tests. Categorical data were presented as n (%) and compared using χ2 tests. Subgroup analyses were conducted based on whether or not patients underwent localization for multiple PNs. P < 0.05 served as the cut-off to define significance, with all analyses being performed with SPSS 16.0 (SPSS Inc., IL, USA).

## Results

### Patients

This study enrolled 121 patients (140 PNs) who underwent IG-based localization and 113 patients (153 PNs) who underwent BSG-based localization (**Table 1**). No significant differences were noted between these groups with respect to age, PN diameter, PN characteristics, PN location, or PN-pleura distance. Significantly more patients in the BSG group required the localization of multiple PNs relative to the IG group (28.3% vs. 13.2%; P = 0.004).

**Table 1:** Baseline data of patients and PNs.

	IG group	BSG group	P value
Patients’ number	121	113	–
Age (y)	50.0 ± 13.4	52.9 ± 9.7	0.053
Gender			0.355
Male	37	41	
Female	84	72	
PNs’ number	140	153	0.004
Patients with 1 PN	105	81	
Patients with multiple PNs	16	32	
Nature of PNs			0.182
Solid	6	14	
Pure GGN	94	91	
Mixed GGN	40	48	
Diameter (mm)	7.1 ± 2.8	7.4 ± 3.1	0.588
PN-pleura distant (mm)	10 (Q1: 4; Q3: 19)	5.5 (Q1: 5; Q3: 17)	0.520
PNs’ locations			0.250
Left upper lobe	38	38	
Left lower lobe	26	21	
Right upper lobe	48	46	
Right middle lobe	8	13	
Right lower lobe	20	35	

BSG, blue-stained glue; GGN, ground glass nodule; IG, indocyanine green; PN, pulmonary nodule.

"-" means not applicable.

### CT-guided localization

CT-guided localization procedures achieved 100% technical success rates in both groups (**Table 2**), with no significant differences between these IG and BSG groups in localization duration (7.2 ± 3.1 min vs. 7.9 ± 2.5 min, P = 0.074) or VAS scores (2.4 ± 0.5 vs. 2.4 ± 0.5, P = 0.787). IG and BSG groups. Pneumothorax affected 8 (6.6%) and 8 (7.1%) patients in the respective IG and BSG groups (P = 0.887), while 12 (9.9%) and 10 (8.8%) patients of these patients experienced pulmonary hemorrhage. None of these complications impacted VATS procedures. IG was less expensive than BSG ($17.2 vs. $165).

**Table 2:** Details of CT-guided localization.

	IG group	BSG group	P value
Technical success of localization	100%	100%	–
Duration of localization (min)	7.2 ± 3.1	7.9 ± 2.5	0.074
Localization-related complications			
Pneumothorax	8 (6.6%)	8 (7.1%)	0.887
Pulmonary haemorrhage	12 (9.9%)	10 (8.8%)	0.780
VAS	2.4 ± 0.5	2.4 ± 0.5	0.787
Cost of the localization material (US dollars)	17.2	165	–

BSG, blue-stained glue; CT, computed tomography; IG, indocyanine green; PN, pulmonary nodule; VAS, visual analog scale.

"-" means not applicable.

### VATS resection

A 100% technical success rate was achieved for VATS sublobar resection procedures in both groups, with no conversion to thoracotomy (**Table 3**). Intraoperative pathological frozen section analyses revealed diagnoses of invasive adenocarcinoma for 11 and 23 PNs in the IG and BSG groups, respectively although only 4 and 18 patients in these groups underwent subsequent lobectomy. For the remaining 12 patients, lobectomy would not have yielded a sufficient pulmonary functional reserve. A total of 55 and 85 PNs in the IG group were resected via wedge and segmental resection procedures, respectively, while 128 and 25 PNs in the BSG group were resected using these respective approaches. The median durations of VATS procedures in the IG and BSG groups were 60 and 92.5 min, respectively (P = 0.001). Final pathological diagnoses for all PNs were consistent with the rapid intraoperative pathological diagnoses.

**Table 3:** Details of VATS.

	IG group	BSG group	P value
PNs’ number	140	153	–
Technical success of sublobar resection	100%	100%	–
Types of VATS			0.001
Wedge resection	51	110	
Segmental resection	85	25	
Wedge resection + subsequent lobectomy	4	18	
Duration of VATS (min)	60 (Q1: 50; Q3: 90)	92.5 (Q1: 62.8; Q3: 125)	0.001
Pathological diagnoses			0.030
Invasive adenocarcinoma	11	23	
Mini-invasive adenocarcinoma	73	55	
Adenocarcinoma *in situ*	32	43	
Precancerous lesion	7	15	
Benign	17	17	

BSG, blue-stained glue; IG, indocyanine green; PN, pulmonary nodule; VATS, video assisted thoracic surgery.

"-" means not applicable.

### Subgroup analyses

In total, multiple PNs were localized in 16 and 32 patients in the IG and BSG groups, respectively (**Table 4**). One-stage localization was performed for multiple PNs in all cases, with technical success rates of 100% in all cases. The duration of localization (10.8 ± 3.5 min vs. 9.5 ± 2.0 min, P = 0.058) and VAS scores (2.8 ± 0.7 vs. 2.5 ± 0.6, P = 0.083) were also comparable between groups. Pneumothorax affected 2 (12.5%) and 5 (15.6%) patients in IG and BSG groups (P = 1.000), whereas 1 (6.3%) and 2 (6.3%) patients in these respective groups experienced pulmonary hemorrhage (P = 1.000).

**Table 4:** Details of CT-guided multiple PNs localization.

	IG group	BSG group	P value
Patients’ number	16	32	–
PNs’ number	35	72	0.530
Patients with 2 PNs	14	24	
Patients with more than 2 PNs	2	8	
Technical success of localization	100%	100%	–
Duration of localization (min)	10.8 ± 3.5	9.5 ± 2.0	0.058
Localization-related complications			
Pneumothorax	2 (12.5%)	5 (15.6%)	1.000
Pulmonary haemorrhage	1 (6.3%)	2 (6.3%)	1.000
VAS	2.8 ± 0.7	2.5 ± 0.6	0.083

BSG, blue-stained glue; CT, computed tomography; IG, indocyanine green; PN, pulmonary nodule; VAS, visual analog scale.

"-" means not applicable.

One-stage resection procedures were performed for multiple PNs in all cases, with a 100% technical success rate. VATS resection types are summarized in **Table 5**. The respective median VATS durations in the IG and BSG groups were 92.5 and 112.5 min (P = 0.061).

**Table 5:** Details of VATS for multiple PNs.

	IG group	BSG group	P value
Technical success of sublobar resection	100%	100%	–
Type of VATS			0.016
All wedge resection	7	20	
Wedge resection + segmental resection	2	9	
All segmental resection	7	2	
Wedge resection + subsequent lobectomy	0	1	
Duration of VATS (min)	92.5 (Q1: 55; Q3: 113.8)	112.5 (Q1: 83.5; Q3: 158.8)	0.061

BSG, blue-stained glue; IG, indocyanine green; PN, pulmonary nodule; VATS, video assisted thoracic surgery.

"-" means not applicable.

## Discussion

A range of solid localization materials have been employed for CT-guided PN localization in recent years, including hook-wire, coil, and radiolabeling materials (7, 8, 13). Several reports, however, have emphasized the advantages of utilizing liquid-based localization materials (14, 15), as they are less expensive, easier to utilize, and associated with higher localization success rates as well as lower rates of complications as compared to solid materials (9, 10, 14).

In their meta-analysis, Wang et al. (9) noted that liquid material-based localization strategies were superior to hook-wire localization with respect to the rates of successful localization (99.8% vs. 96.0%, P = 0.002) and complications (28.7% vs. 37.8%, P = 0.007). In a separate report, Wang et al. (10) determined that the localization success rates associated with MB were higher than those for coil localization, with a corresponding reduction in rates of postoperative complications. The higher rates of technical failure for localization procedures performed using coil and hook-wire approaches are primarily attributable to the potential for the dislodgement of these materials (9, 10). The rigid nature of these solid materials may also contribute to the higher rates of localization-related complications associated with their use (9, 10).

Here, the safety and efficacy of CT-guided localization procedures performed using two different liquid materials (IG and BSG) were compared, with a 100% rate of successful localization having been achieved in both groups without any difference in the duration of localization. BSG is a localization material prepared in-house that is superior to MB and medical glue. Medical glue-based localization necessitates the detection of this material via palpation during VATS procedures owing to its hyaline nature (4), whereas BSG can be visualized directly owing to its coloration. The coagulable nature of medical glue can also mitigate MB diffusion, making it realistic to achieve a 100% success rate when performing BSG-based localization.

IG-based localization depends strongly on effectively controlling the IG volume (14, 15). Injecting excessive liquid volumes can result in the overflow of these materials, while a volume of ~0.3 mL is reportedly sufficient (14, 15). Here, 0.15 ml of IG was injected into these patients and this was sufficient to achieve effective localization. This strategy also enabled smooth IG injection into these patients, which can also protect against the risk of overflow associated with more rapid injection.

Similar localization-associated pulmonary hemorrhage and pneumothorax rates were noted in both groups, suggesting that IG- and BSG-based localization procedures are similarly safe. Notably, these rates of pneumothorax (IG: 6.6%; BSG: 7.1%) and pulmonary hemorrhage (IG: 9.9%; BSG: 8.8%) were both reduced as compared to prior reports focused on PN localization procedures performed with hook-wire (pneumothorax: 37.8%; pulmonary hemorrhage: 24.3%) or coil (pneumothorax: 18.9%; pulmonary hemorrhage: 27.3%) approaches (4, 16). These liquid-based materials may thus be safer than solid materials in this context. The needle required for liquid material injection is also relatively small, further contributing to potential reductions in adverse complication rates.

VATS sublobar resection was successfully utilized to remove the target nodules in all cases in the present study, suggesting that localization material has no impact on VATS procedural performance. However, the IG group did exhibit a significant reduction in VATS duration relative to the BSG group (60 vs. 92.5 min, P = 0.001). This may be attributable to the fact that more patients in the BSG group required the localization of multiple PNs and that subsequent lobectomy was required by more patients in the BSG group following sublobar resection relative to the IG group. Both of these factors can prolong the VATS procedure.

Subgroup analyses were additionally conducted based on patients requiring the localization of multiple PNs. In these analyses, both IG and BSG localization strategies remain comparable with respect to the technical success rates for both localization and VATS procedures, the duration of localization, the duration of VATS procedures, and the incidence of localization-associated complications. As such, both IG and BSG can be effectively used to localize multiple PNs. Preoperative localization is essential for patients harboring multiple PNs, as it can enable effective one-stage VATS sublobar resection procedures (17). Indeed, most patients in this subgroup only needed to undergo multiple sublobar resection procedures, thus maximizing the preservation of respiratory function in these individuals.

An important limitation to note is that this is a retrospective analysis subject to a high risk of bias, emphasizing a need for subsequent randomized controlled trial-based validation. Moreover, the experience and skill levels of the operators from the two different participating hospitals were not identical, potentially introducing additional bias, although technical success rates of 100% were achieved for localization and VATS procedures in all cases. Lastly, the number of enrolled patients with multiple PNs was relatively small such that results related to multiple PN localization are less likely to be reliable, underscoring a need for further analyses with a larger sample size.

## Conclusion

These data reveal that the efficacy and safety of IG- and BSG-based CT-guided localization are comparable for patients with PNs, although the use of IG is less expensive than that of BSG.

## Data availability statement

The original contributions presented in the study are included in the article/supplementary material. Further inquiries can be directed to the corresponding authors.

## Ethics statement

The studies involving humans were approved by Ethics Committee of The First Affiliated Hospital of Ningbo University and Ethics Committee of Anhui Provincial Hospital. The studies were conducted in accordance with the local legislation and institutional requirements. The requirement for informed consent was waived because this study is a retrospective study.

## Author contributions

JL: Conceptualization, Writing – original draft. JZ: Formal analysis, Writing – original draft. NW: Methodology, Writing – review & editing. A-LW: Supervision, Writing – review & editing. L-FW: Data curation, Writing – review & editing. FT: Methodology, Writing – review & editing. Y-TX: Data curation, Writing – review & editing. RH: Data curation, Writing – review & editing.
